# Coumarins as Tool Compounds to Aid the Discovery of Selective Function Modulators of Steroid Hormone Binding Proteins

**DOI:** 10.3390/molecules26175142

**Published:** 2021-08-25

**Authors:** Sanna Niinivehmas, Olli T. Pentikäinen

**Affiliations:** 1 Faculty of Medicine, Institute of Biomedicine, University of Turku, FI-20520 Turku, Finland; 2 InFLAMES Research Flagship Center, University of Turku, FI-20520 Turku, Finland; 3 Aurlide Ltd., FI-20520 Turku, Finland

**Keywords:** coumarin, 3-phenylcoumarin, estrogen receptor, aromatase, 17β-hydroxysteroid dehydrogenase

## Abstract

Steroid hormones play an essential role in a wide variety of actions in the body, such as in metabolism, inflammation, initiating and maintaining sexual differentiation and reproduction, immune functions, and stress response. Androgen, aromatase, and sulfatase pathway enzymes and nuclear receptors are responsible for steroid biosynthesis and sensing steroid hormones. Changes in steroid homeostasis are associated with many endocrine diseases. Thus, the discovery and development of novel drug candidates require a detailed understanding of the small molecule structure–activity relationship with enzymes and receptors participating in steroid hormone synthesis, signaling, and metabolism. Here, we show that simple coumarin derivatives can be employed to build cost-efficiently a set of molecules that derive essential features that enable easy discovery of selective and high-affinity molecules to target proteins. In addition, these compounds are also potent tool molecules to study the metabolism of any small molecule.

## 1. Introduction

Coumarins form a versatile group of both naturally and synthetically occurring molecules. In nature, coumarins are found in a wide variety of plants, with exceptionally high concentrations in tonka bean (*Dipteryx odorata*) from which it was first isolated. The structural and physicochemical properties of coumarins make them a multipurpose scaffold in drug design, medicinal chemistry, and chemical biology. The simple chemical backbone and the reactivity of the conjugated double ring system of coumarins (an α-pyrone ring fused with a benzene ring) make them exciting molecules for different research fields. In addition to simplicity, coumarins have several attractive features, such as low molecular weight, high bioavailability, high solubility in most of the organic solvents, and low toxicity [1,2,3].

Coumarins can be found, for example, in cosmetics and perfumes, as food additives, in rat poison, and especially in the products of the pharmaceutical industry. Coumarins have their place in biomedicine in drug discovery projects and fluorescent probes in imaging and assay development. Coumarins exhibit several pharmacological effects, including anticancer, anticoagulant, antimicrobial, antiviral, anti-inflammatory, antioxidant, neuroprotective, fungicide, antidiabetic, anticonvulsant, and antiproliferative activities. Pharmacological and biological applications of coumarin derivatives are variable; thus, also their protein targets and measured activities range significantly [1,2,3,4].

**Figure 1:**
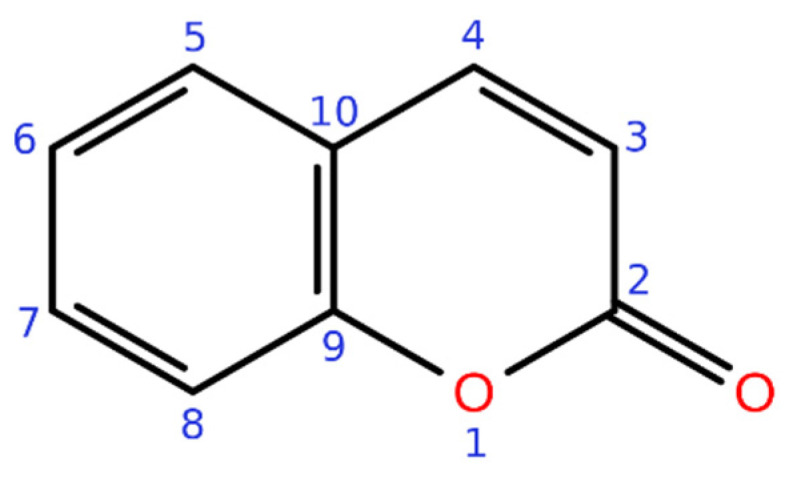
Coumarin.

**Figure 2:**
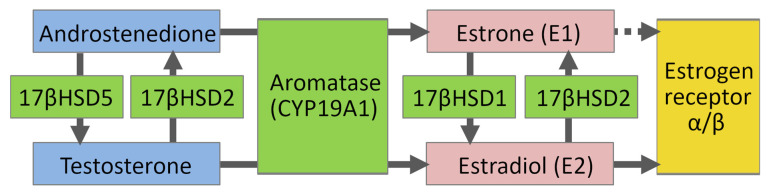
Steroid synthesis pathway.

Natural coumarins can be subdivided into different classes based on their chemical structures; simple coumarins (Figure 1), isocoumarins, furanocoumarins, and pyranocoumarins (both angular and linear), biscoumarins, and other coumarins such as phenylcoumarins [2,5]. This review concentrates on 3-phenylcoumarins and their application as steroid mimics in steroid hormone biosynthesis pathways (Figure 2). 

In the androgen pathway, testosterone is formed by 17β-hydroxysteroid dehydrogenase type 5 (HSD5) from androstenedione (Figure 2). Vice versa, the conversion of testosterone to androstenedione is catalyzed in oxidation by 17β-hydroxysteroid dehydrogenase type 2 (HSD2). In the aromatase pathway, androstenedione is converted to estrogen (E1) and testosterone to the biologically most active estrogen, 17β-estradiol (E2), by the aromatase (CYP19A1). In the latter part of the sulfatase pathway, E1 is converted to E2 in reduction by 17β-hydroxysteroid dehydrogenase type 1 (HSD1). Vice versa, the conversion of E2 to less active E1 is catalyzed in oxidation by HSD2. Estrogenic response takes place when E2 binds and activates estrogen receptors (ER) [6]. This review focuses on estrogen receptor α (ERα), HSD1, HSD2, and aromatase. Finally, other applications and targets of 3-phenylcoumarins are touched upon.

## 2. Discussion

**Figure 3:**
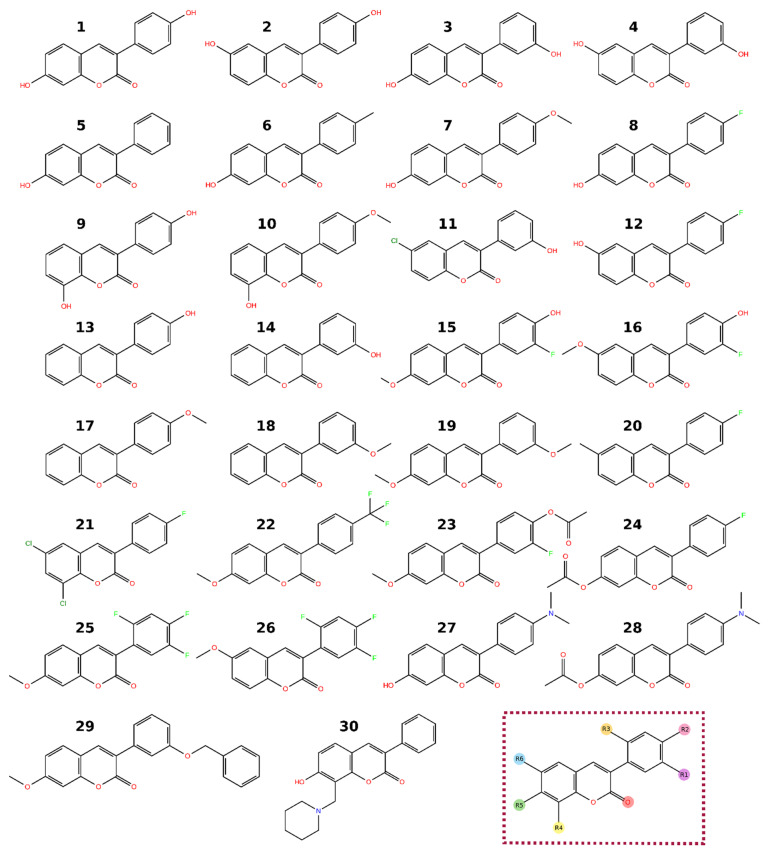
Structures of the studied 3-phenylcoumarin derivatives.

The applicability of easy-to-synthesize 3-phenylcoumarin derivatives as steroid mimics in steroid hormone biosynthesis pathways was analyzed. These compounds are synthesizable with microwave-assisted organic synthesis in few minutes from cheap starting materials with one-step synthesis (excluding possible protecting groups) [7,8,9]. The 3-phenylcoumarin ring system is expected to adopt similar hydrophobic packing at the active site of steroid hormone enzymes and receptors as the established steroidal compounds. Moreover, several polar substituents (mainly hydroxyl, methoxy, or halogen) were introduced to the 3-phenyl ring R1–R3 positions and the coumarin ring R4-R6 positions to enable strong binding interactions (Figure 3; Table 1).

### 2.1. Estrogen Receptor α

ER is a nuclear hormone receptor, which mediates E2 action in different parts of the body. ER is an established target for drug development, e.g., in endocrine-based breast cancer therapy and menopausal hormone replacement therapy. A clear majority of breast cancer tumors are ER-positive, and tumor growth is linked to high E2 levels promoting ER activity and/or an increased number of ERs. Among ER binding ligands, selective estrogen receptor modulators (SERM) are nowadays widely used, as they allow selective inhibition or stimulation of E2 action in various tissues [10,11].

It has been shown that 3-phenylcoumarins can mimic steroid compound binding in ER and thus offer a solution to affect ER activity [7].

**Figure 4:**
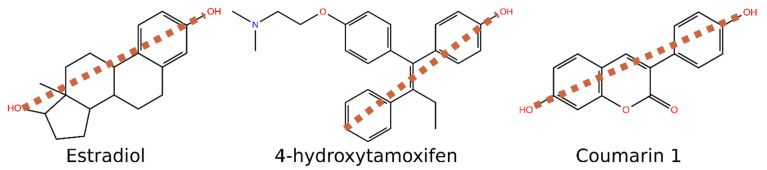
Simple 2D molecular topology pharmacophore for ER binders. Estradiol, 4-hydroxytamoxifen, and 3-phenylcoumarin core divide equally when a line is drawn from the A-ring/3-phenyl ring to the other end of the compound.

**Figure 5:**
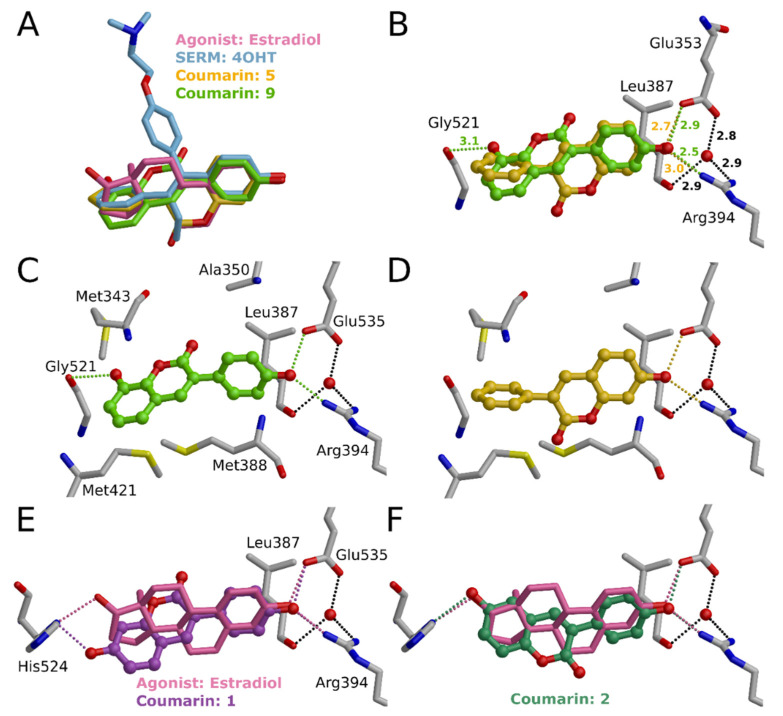
Binding of 3-phenylcoumarins to ERα. (**A**) Comparison of possible binding conformations of estradiol (PDB: 1ERE [12]), 4-hydroxytamoxifen (4OHT; PDB: 3ERT [13]) and two 3-phenylcoumarins **5** and **9** at the binding site of ERα. (**B**–**D**) Comparison of possible binding modes of 3-phenylcoumarins with hydroxyl either in R2 (**9**) or in R5 (**5**) position. Different hydroxyl substituent position makes coumarin core to flip so that favorable interactions with Glu353 and Arg394 are formed. (**E**,**F**) Comparison of 3-phenylcoumarins having two hydroxyl substituents. The most obvious estradiol mimic, compound **1,** approaches His524 at a different angle compared to D-ring hydroxyl of estradiol, whereas compound **2** forms a similar hydrogen-bonding network as estradiol.

Practically all developed small molecule ER agonists, partial agonists, and antagonists share the same molecular topology (Figure 4) that inherits from E2. Accordingly, it is not a big surprise that coumarin derivatives bind to ER. By simply varying the position of polar groups, it is easy to derive topological pharmacophore for binding. It is possible to draw a line from the 3-hydroxyl group of E2 to 17-hydroxyl of E2 and see that the steroid core is almost equally divided above and below the line (Figure 4). Similarly, the traditional, active form of breast cancer drug 4-hydroxytamoxifen has such a feature. When checking the validity of such a simple pharmacophore, we studied the binding of various 3-phenylcoumarin derivatives. Basically, all active coumarin derivatives share the same simple pharmacophore and have a phenol group that mimics phenolic A-rings of E2 and 4-hydroxytamoxifen (Figure 4). In addition to 2D pharmacophore, the same phenomenon can be visualized in the 3D overlay of estradiol, 4-hydroxytamoxifen and 3-phenylcoumarin (Figure 5A).

**Figure 6:**
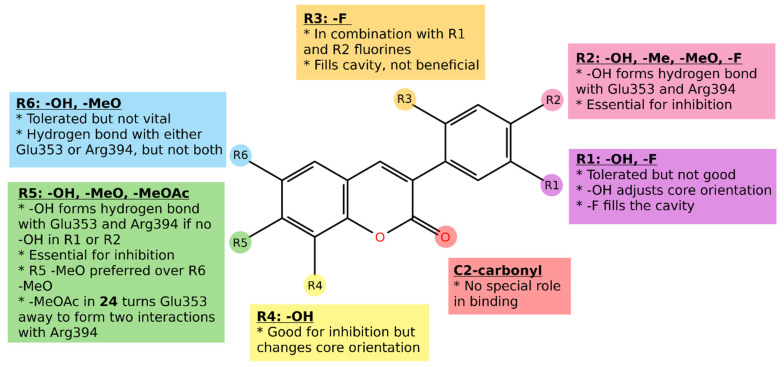
The docking-based structure–activity relationship analysis of the 3-phenylcoumarin derivatives with ERα. Although C2-carbonyl of the coumarin core does not form direct interactions in the ERα binding site, it helps in maintaining the planar geometry of the compounds.

For the ERα binding cavity, coumarin derivatives have the correct size. However, the main reason why these compounds are suitable for ERα is phenolic hydroxyl as a functional group. Eleven compounds show higher than 55% inhibition against ERα in 10 μM concentration (**1**–**2**, **5**–**9**, **13**, **15**–**16**, **24**; Table 1). Five of the active compounds (**2**, **9**, **13**, **15**–**16**; See **2** and **9** in Figure 5) have hydroxyl in the 3-phenyl ring in the R2 position (Figure 3). Similarly, five of the active compounds (**1**, **5**–**8**; See **1** and **5** in Figure 5) have a hydroxyl group in the R5 position (Figure 3). This indicates that both the 4′-hydroxy-3-phenylcoumarin and 7-hydroxycoumarin core seem to be an excellent basis for the design of ERα binders. These phenolic hydroxyl groups (R2 or R5 position; Figure 6) form a strong, attractive hydrogen bonding network with Glu353, Arg394, and a water molecule (Figure 5B–D).

Three of the active compounds (**1**–**2**, **9**) have two hydroxyl substituents, thus acting as the clearest mimics for estradiol. Interestingly, compound **1** has lower activity than the other two, which is also the most like estradiol structurally. There is a moderate change in the angle, at which R5 hydroxyl approaches His524 when compared to D-ring hydroxyl of estradiol (Figure 5E). For comparison, compound **2**, which has hydroxyl in the R6 position, can form hydrogen-bonding network comparable to estradiol (Figure 5F). In general, ERα activity is sensitive to the number of hydroxyls and their placement in the 3-phenylcoumarin core.

Of the most active compounds, **24** does not have any hydroxyl substituents, making it different from other active compounds. Consequently, **24** also has an aberrant binding mode: 7-methyl acetate could shift Glu353 away, giving substituent space to form tight double interaction with Arg394, boosting binding affinity. Glu353 is not left alone but forms interactions with the main chain oxygen of Leu327 and via a water molecule with the main chain oxygen of Pro325.

The hydroxyl group in the R6 position is also tolerated. R6 position hydroxyl in **2** can form a well-coordinated hydrogen bond with His524 or Gly521, whereas R1 position hydroxyl in **4** changes core orientation slightly, but so that interactions are not in optimal angle; hence the only low activity (5-fold difference in inhibition percentage of **2** and **4**; Table 1). This modification does not change the location of the coumarin core in the binding site drastically; however, **4** can form only one hydrogen bond with Glu535. Similarly, as **4**, also **3** and **14** have a hydroxyl group in the R1 position, and this positioning of the hydroxyl is not optimal and thus either shifts or flips the coumarin core diminishing the activity. **12** has the same favorable hydroxyl group (R6 position) as **2** and **4**, but missing the other hydroxyl substituent and having fluorine in the R2 position instead flips the compound. Possible halogen bonding with R2 fluorine is too directional, thus making the **12** too stiff for the cavity. This leads to a clash with His524, which forces the shift in coumarin core-binding mode, thus diminishing the activity.

Other positions for the hydroxyl group are tolerated as well. The hydroxyl group in the R4 position of **9** yields high activity, at least in combination with R2 hydroxyl (Figure 6). While R2 hydroxyl binds with Glu353 and Arg394, R4 hydroxyl can form a well-coordinated hydrogen bond with His524 or Gly521. In the same way, **10** has R4 hydroxyl, but R2 position methoxy cannot form vital interactions, as R2 position hydroxyl in **9** can, but collides with important Glu353, Arg394, and a water molecule site; hence the molecule is inactive.

Decent ERα binders can also be built by adding other substituents than hydroxyl in the 3-phenyl ring, such as in **6**–**8** (Figure 3; Table 1). **15**–**16** have a beneficial hydroxyl group in the R2 position. Fluorine next to the hydroxyl in the R1 position is as well tolerated and fills the cavity. In these compounds, the R5 position methoxy is more favorable than the R6 position methoxy. For example, R5 methoxy packs into hydrophobic surroundings created by Met421, Ile424, Gly521, and His524, whereas for R6-methoxy, hydrophobic packing is looser, or binding requires coumarin core to flip, which can push the compound to the side of the binding cavity, leaving an unfavorable, hollow area to the other side of the ligand. However, these compounds are a bit on the large side to fit the binding site of ERα. Equally, the same explanation suits **25** and **26**, having R5 and R6 methoxy substituent, respectively, although these compounds with R1-R3 fluorines are inactive. The remaining compounds either do not have an important hydroxyl group (**17**–**18**, **20**, **31**) and, in addition, can be too large (**19**, **21**–**22**).

### 2.2. 17β-Hydroxysteroid Dehydrogenase Type 1

HSD1 overexpression is a strong signal for, e.g., breast cancer and endometriosis; whereas, HSD2 is known to have an inhibitory effect in breast tumorigenesis. HSD1 converts E1 to E2, promoting high E2 activity, while HSD2 acts conversely [14]. A considerable number of both steroidal and non-steroidal HSD1 inhibitors have been published due to screening campaigns and rational drug design approaches; however, only very few compounds have been applied in vivo in preclinical studies, and none has passed clinical trials so far [15]. Similarly, as with ER, coumarin derivatives can mimic estrogen binding in the active site of HSD1 by imitating hydrophobic packing of the steroid ring. 3-phenylcoumarin has proven to be a suitable non-steroidal scaffold for building small-molecule inhibitors targeting HSD1 [8].

**Figure 7:**
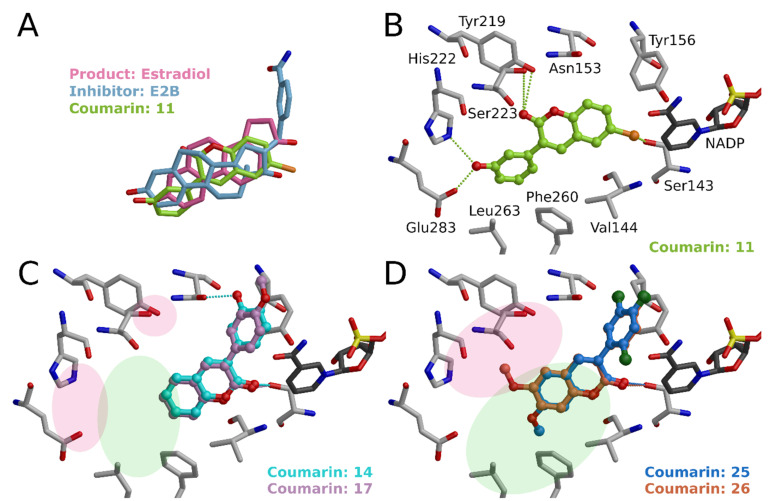
Binding of 3-phenylcoumarins to HSD1. (**A**) Comparison of possible binding conformations of estradiol (PDB: 1A27 [16]), E2B inhibitor (PDB: 3HB5 [17]), and 3-phenylcoumarin **11** at the binding site of HSD1. (**B**) General binding mode of 3-phenylcoumarins at the binding site of HSD1 represented by **11**. 3-phenyl ring hydroxyl substituent (R1, R2; here R1-OH) binds with His222 and Glu283. Coumarin core substituent (R5, R6; here R6-Cl) bind with catalytic Ser143 and/or Tyr156. C2-carbonyl of the coumarin core binds with Tyr219 and/or Ser233. (**C**) One of the most active compounds, **14**, has a different binding mode. R1 position hydroxyl interacts with Asn153 and C2-carbonyl with Ser143. In addition, the hydrophobic packing of this compound is excellent. **14** would offer an excellent skeleton to build a novel HSD1 inhibitor by growing a hydrophobic core (light green area) and adding potential hydrogen bond-forming groups (red area). **17** demonstrates that a similar effect is not achieved with, e.g., R2 methoxy. (**D**) Comparison of 3-phenylcoumarins that have methoxy either in R5 (**25**) or in R6 (**26**) position. Different substituent position shows that favorable R5 position methoxy can stack with, e.g., Phe260 and Leu263 (green area) whereas methoxy in R6 position ends up to unfavorably close to Tyr219, His222, and Ser223.

**Figure 8:**
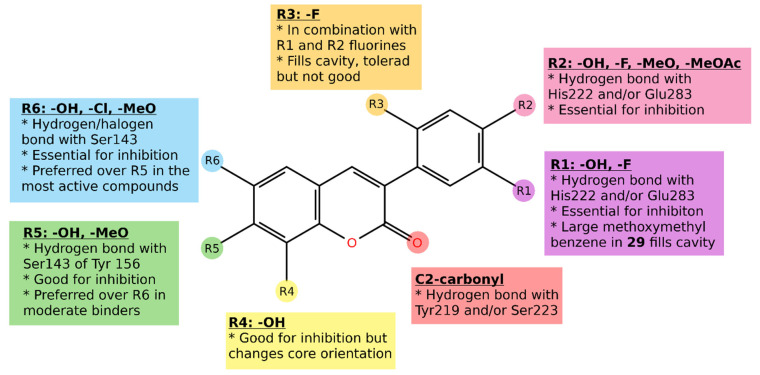
The docking-based structure–activity relationship analysis of the 3-phenylcoumarin derivatives with HSD1.

Three of the best derivatives produced 68% inhibition at 1 μM (**2**, **10**–**11**, Table 1; See **11** in Figure 7). Altogether twelve compounds showed decent inhibition at 1 μM. Even at the 100 nM level, one of the compounds, **11**, produced 47% inhibition [8]. The best compounds are dual hydroxyls (**1**–**2**, **4**) or are otherwise able to accept and/or donate a hydrogen bond in both ends of the compound (**10**–**12**, **15**–**16**) (Figure 3 and Figure 8). These compounds can form hydrogen bonds with the residues lining both ends of the binding cavity; in the catalytic site with catalytic Ser143 and nearby Tyr156 and in the other end with His222 and Glu283 (Figure 7B). In the best case, Ser223 and Tyr219 in the middle of the binding cavity nail the ligand to its place by binding to the carbonyl of coumarin core. In addition, hydrophobic residues Val144, and Leu150, Pro188, Val226, Phe227, Phe260, Leu263, and Val284 finalize the network of favorable interactions.

Of the most active compounds, **14** has an aberrant binding mode (Figure 7C). Hydroxyl group interacts with Asn153 and coumarin core carbonyl with Ser143. In addition, 3-phenyl ring stacks perfectly with Tyr156 and Phe193 and hydrophobic parts of the coumarin core stack with Phe160, Pro188, Val144, and Leu150. This compound would offer an excellent skeleton to build a novel HSD1 inhibitor (Figure 7C). **13** has one hydroxyl group as **14** and thus can only form interactions in one end; however, **13** with R2 position hydroxyl cannot adopt the same aberrant binding mode as **14**, which hydroxyl is in R1 position, and then favorable interactions are lost.

Analogs **6**–**8**, **27** have variable substituents in the R2 position that are not as favorable as hydroxyl in **1**. If we compare, for example, **1** and **7**, the methyl in methoxy cause steric repulsions that affect both binding angles and causing a collision when binding. Again, if we compare **8** with **1**, they both have a hydroxyl substituent (R5 position) and fluorine or hydroxyl (R2 position), respectively. Fluorine in the R2 position creates a potential for halogen bonding; however, the directionality of bond formation is wrong, whereas for hydroxyl angle is much better. These examples indicate the importance and effect of well-coordinated hydrogen/halogen bonds.

Less active compounds rely mainly on hydrophobic interactions (**17**–**22**, **24**–**26**, **28**, **30**; see **17**, in Figure 7C, and **25** and **26** in Figure 7D). Generally, they typically have a limited ability to form hydrogen bonds; typically, they form only one hydrogen bond by carbonyl of coumarin core. They can adopt different binding modes compared to active compounds. Here carbonyl of the coumarin core interacts with catalytic Ser143, and hydrophobic coumarin core is surrounded by Phe260, Pro188, Leu150, and Val144. Similarly, the 3-phenyl ring is clamped between rings of Phe227 and Tyr156. This mode is likely adopted to avoid the area of His222 and Glu283 and to benefit from hydrophobic surroundings. For example, if we compare **25** and **26** (Figure 7D), methoxy of **25** in R5 position can stack with Phe260 and Leu263 whereas methoxy of **26** in R6 position ends up to unfavorable environment close to His222, and this both explains the difference in the activities and highlights the importance of formation of optimal hydrophobic interactions.

Larger compounds **23** and **29** showing modest activity can somewhat compensate for their lack of hydrogen bond donors and acceptors with additional hydrophobic interactions. In summary, less active compounds do not have enough hydrogen bond donors/acceptors to form favorable interactions, or their substituent is methoxy, which is sterically hindered by its own methyl group and cannot coordinate as many or as strong interactions as, e.g., hydroxyl.

### 2.3. 17β-Hydroxysteroid Dehydrogenase Type 2

HSD2 is the enzymatic counterpart for HSD1. Therefore, to avoid the contradictory effect, it is crucial that any potential drug aiming to lower the E2 production should not notably block the HSD2 activity [14,15]. Unfortunately, only some of the compounds have been experimentally tested for HSD2 binding. Of the tested compounds, most are only modestly active with HSD2, which is, as mentioned, a desirable quality. The activity measurements show that none of the 3-phenylcoumarin analogs produce > 50% HSD2 inhibition at 1 μM (Table 1). **2** is the most selective dual ERα and HSD1 inhibitor with only 7% HSD2 inhibition. The most potent HSD1 inhibitor analog, **11**, blocks the HSD2 only faintly (16%; Table 1). Irritatingly, there is no 3D structural data on HSD2, and thus binding of the 3-phenylcoumarin derivatives cannot be visualized.

### 2.4. Aromatase

**Figure 9:**
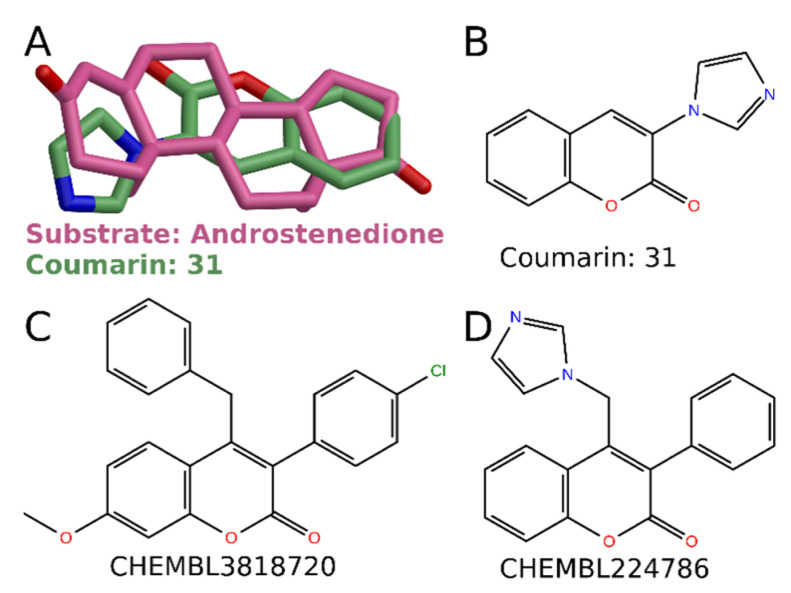
Aromatase inhibitors with a 3-phenylcoumarin core. (**A**) Comparison of possible binding conformations of androstenedione (PDB: 3EQM [22]) and 3-phenylcoumarin **31** at the binding site of aromatase. 2D structures of (**B**) coumarin 31, (**C**) CHEMBL3818720 [20] and (**D**) CHEMBL224786 [21].

Aromatase (cytochrome P450 (CYP) 19A1) catalyzes the conversion of androstenedione to E1 and testosterone to E2, being the only vertebrate enzyme capable of catalyzing the aromatization of a six-membered ring [18]. Aromatase inhibitors are primarily used by post-menopausal patients having estrogen-dependent breast cancer because the E2 concentration in breast carcinoma tissue is locally higher than elsewhere in the body [19]. Although the 3-phenylcoumarin scaffold mimics the steroid core (Figure 9A) and fits into the active site of the aromatase, experimentally tested analogs do not inhibit aromatase. The polar substituent in the 3-phenylcoumarin analogs lacks favorable interactions at the active site of aromatase.

**Figure 10:**
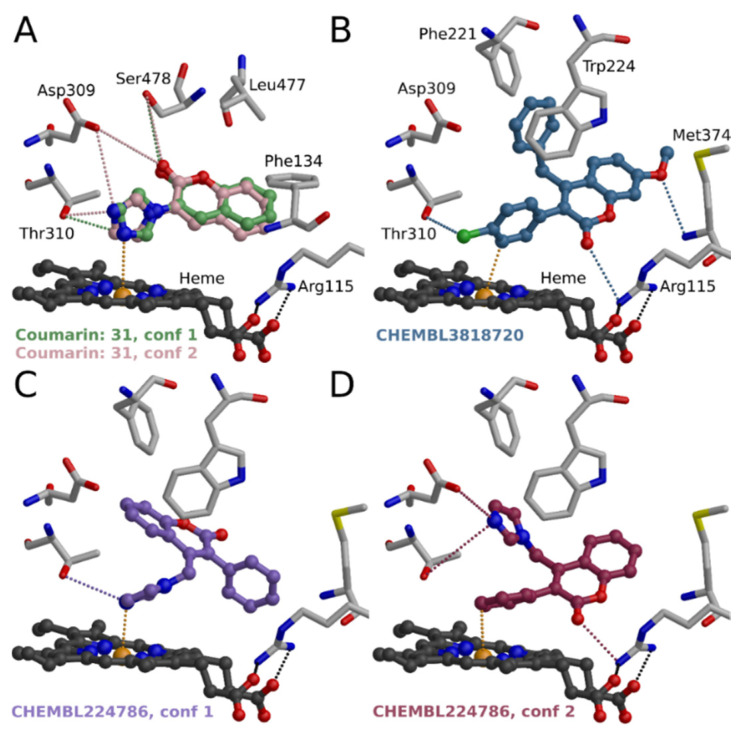
Binding of compound **31** and 3-phenylcoumarins to aromatase. (**A**) Comparison of possible binding conformations of **31** at the binding site of aromatase (PDB: 3EQM [22]). Nitrogen in the 3-imidazole ring of **31** coordinates either directly with heme (conformation 1) or forms hydrogen bonds with Asp309 and/or Thr310 (conformation 2). (**B**) Possible binding mode of CHEMBL3818720 [20]. The 3-phenyl ring could coordinate with heme. Possible binding modes of CHEMBL224786 [21] so either (**C**) imidazole ring or (**D**) 3-phenyl ring coordinates with heme.

However, a simple hydrogen bond acceptor at the R1 or R6 position (such as a carbonyl group in androstenedione, Figure 9A) would be needed to avoid the unfavorable clash of proton donors at the active site. This observation led to the synthesis and discovery of 3-imidazolecoumarin as a potent aromatase inhibitor **31** (Figure 9B). Nitrogen in the 3-imidazole ring of **31** could coordinate either directly with heme (conformation 1 in Figure 10A) or form hydrogen bonds with Asp309 and/or Thr310 (conformation 2 in Figure 10A). C2-carbonyl of the coumarin core could form a hydrogen bond with Asp309 and/or Ser478. In addition, hydrophobic coumarin core is surrounded very favorably with Ile 133, Phe143, Phe221, Trp224, Val370, Leu372, Met374, and Leu477. The activity measurements show that **31** strongly inhibits the aromatase (pIC_50_ 7.1 [8]). Moreover, cross-reactivity testing indicates that **31** does not inhibit other tested targets [8].

In the literature, there are some published 3-phenylcoumarin inhibitors for aromatase. Chen et al. (2004) studied series of 21 coumarin derivatives and found that 4-benzyl-3(4′-chlorophenyl)-7-methoxycoumarin (CHEMBL8318720; Figure 9C) is a significantly more potent inhibitor of aromatase than several known aromatase inhibitors (K_i_ 84 nM) [20]. Leonetti et al. (2004) published aromatase inhibitors bearing either an imidazole or a triazole ring linked to a fluorene, indenodiazine, or coumarin scaffold [21]. In general, coumarin was described as the best core, and one of the reported compounds was a 3-phenylcoumarin: 4-(imidazol-1-ylmethyl)-3-phenylchromen-2-one (CHEMBL224786; compound **31** in [21]; Figure 9D) showed some potency as an aromatase inhibitor (pIC_50_ 5.3).

### 2.5. Previously Studied 3-Phenylcoumarins

Coumarins are broadly studied compounds with great structural and pharmacological variability. Thus, it is not surprising that also 3-phenylcoumarins have been studied elsewhere against both the same targets discussed here and also a wide range of other targets. In PubMed search with keywords 3-phenylcoumarin or 3-arylcoumarin altogether, 70 articles were found (12 July 2021). In ChEMBL database [23,24] 3-phenylcoumarin structure search produced 896 different compounds, with a total of 7608 measured activities (18 June 2021). One of the most popular tested targets is ERα, to which 108 distinct 3-phenylcoumarin compounds were reported. 

Compound **1** has been tested by Yang et al. (2017), who found out that it did not produce an effect on the antagonist mode of ERα but produced an effect in the agonist mode of ERβ (CHEMBL1915315; compound **3a** in [25]). They also found out that the introduction of a larger substituent than methyl group at the 4-position of coumarin core in addition to 3-phenyl ring caused a major boost on the binding affinity of ERs. In the 3-phenyl ring, they found out that additional *ortho* substitution was better than the corresponding unsubstituted compound **1**, whereas added *meta* substitution dramatically decreased binding affinity [25]. Similarly, Shen et al. (2010) showed that **13** (CHEMBL71271; compound **2f** in [26]) has agonist activity for both ERα and ERβ, but with great selectivity towards ERβ, which could be exploited in molecular probes to differentiate the biological roles of the two subtypes [26].

It is known that monoamine oxidases A and B (MAO-A, MAO-B) are inhibited to some degree by the 3-phenylcoumarin analogs, and thus quite a number of compounds are presented here, have been studied against MAOs. The inhibitory effect is especially noteworthy for MAO-B (see, e.g., [9,27,28,29,30,31]). Comparably to earlier studies, coumarin derivatives that inhibit HSD1 (1 μM) also tend to inhibit MAO-B (10 μM) [8,9]. Of the HSD1 inhibitor analogues **15**–**16**, **18**, **20**, **22**–**23**, **26**, **28** have IC50 < 1 μM against MAO-B [9]. For compounds **2**, **4**, **10**, **29,** inhibition of HSD1 at 1 μM is higher than for MAO-B at 10 μM [9]. Especially noteworthy is that the best dual ERα/HSD1 inhibitor **2** could be selective over MAO-B, similarly to HSD1 inhibitor **10**. The only compound that inhibits MAO-A at a moderate level is **6**; however, the inhibition is under 50% at a relatively high 100 μM concentration [9]. **11** (compound **6** in [27]) and **17** (compound **3d** in [28]) have been tested for MAO-A and MAO-B and confirmed clearly selective towards MAO-B. **1**–**2**, **5**–**10**, **13** (compounds **4**, **11**, **1**, **16**, **13**, **18**, **6**, **14**, **5**; respectively in [29]) have been tested for ability to inhibit MAO-B, acetylcholinesterase and butyrylcholinesterase.

Most estrogens are metabolized first in the liver by CYP enzymes. In this metabolism, the CYP1A2 enzyme has an important role [32] and, thus, the unintended inhibition could induce an increase in E2 levels. Virtually all tested 3-phenylcoumarins block CYP1A2 activity at some concentration [9]. However, for the best three HSD1 inhibitors, **2**, **10**, **11**, only the most potent HSD1 inhibitor **11** blocks CYP1A2 at an alarming level [8]. 3-phenylcoumarins have been widely studied as profluorescent substrates for variety of CYP enzymes: compounds **2**, **8**, **11**, **18**–**19**, **29** (compounds **7**, **12**, **4**, **8**–**10**; respectively in [33]) and **4**, **14**, **16**, **20** (compounds **15**, **23**, **13**, **21**; respectively in [34]). A wide panel of studied CYP enzymes and 3-phenylcoumarin compounds show that these compounds have the potential to act as tool molecules when the metabolism of small molecules is under investigation.

Some of the compounds discussed here have been previously published for inhibitory activity against completely other targets. **1**, **4**–**10**, **13** (compounds **12**, **17**, **4**, **9**, **24**, **18**, **14**, **22**, **6**; respectively in [35]) have been investigated for antidiabetic activity, i.e., tested for antioxidant, α-glucosidase inhibitory, and advanced glycation end-products formation inhibitory activity. **2** (compound **13**) and **4** (compound **11**) have been tested for inhibition of horseradish peroxidase [36]. **2** (compound **4**) and **13** (compound **3**) have been studied as potential inhibitors of mast cell degranulation, a key event for the development of allergic reactions [37]. **5** and **9** (CHEMBL153505, 3-phenylumbelliferone or compound D, and CHEMBL1777848, compound **11**; respectively in [38]) have been tested for inhibition of tyrosinase. **5** has shown promise also as a tautomerase inhibitor of macrophage migration inhibitory factor (CHEMBL153505; compound **8** in [39] and compound 3 in [40]). In addition, **5** (compound **9** in [41]) has been tested for antibacterial activity. **10** has shown activity against HIV-1 replication (compound **17** in [42]). **13** and **14** (CHEMBL71271, compound **19** and CHEMBL71407, compound **18**; respectively in [43]) have shown moderate inhibitory activity against glyceraldehyde-3-phosphate dehydrogenase.

Several 3-phenylcoumarins have shown the ability to reduce oxidative stress and thus having shown anti-inflammatory activity: **1**, **5** and **13** (CHEMBL1915315, compound 3, CHEMBL153505, compound **1**, and CHEMBL71271, compound **2**; respectively in [44]), **2** (CHEMBL486894, compound **13** in [45]), **4** (CHEMBL472548, compound 11 in [45]/compound **2** in [46]), **9** (CHEMBL1777848, 9 in [47]), and **17** and **18** (CHEMBL1714497, compound **20**; CHEMBL3359868, compound **19**; respectively in [48]).

Coumarins have also been utilized as excellent probes for different assays and imaging. For example, **5** (CHEMBL153505; compound **12**), **27** (CHEMBL1765812; compound **30**), and **28** (CHEMBL1765811; compound **29**) have shown potential for employment as molecular probes for imaging of myelination [49].

### 2.6. Other Targets for Other 3-Phenylcoumarins

Demkowicz et al. (2016) performed the synthesis and biological evaluation of fluorinated 3-phenylcoumarin-7-*O*-sulfamate derivatives as steroid sulfatase inhibitors [50]. Steroid sulfatase acts in the earlier phases of the sulfatase pathway converting estrone sulfate to E1. Inhibition of this enzyme gives one additional proof of the ability of coumarins to mimic steroid core in ligand binding area of different enzymes and receptors.

3-phenylcoumarins are utilized widely in the biomedical and pharmaceutical industry. 3-phenylcoumarins bearing aminoalkoxy moiety has been designed to treat Alzheimer’s disease by inhibiting acetylcholinesterase and butyrylcholinesterase [51]. 3-phenylcoumarins with dihydroxyl substituents in the coumarin core have been studied for antioxidative effect [52,53]. Radioiodinated 3-phenylcoumarins have been evaluated targeting myelin in multiple sclerosis [54]. Simple 3-phenylcoumarins may serve as potential antidepressant agents [55]. 3-phenylcoumarins act as carriers for potent antibacterial agents against, e.g., methicillin-resistant *Staphylococcus aureus* [56] or mycotoxigenic fungus *Aspergillus flavus* [57]. 3-phenylcoumarins have been shown to modulate several protein targets, e.g., glutathione S-transferase [58], Hsp90 [59], and human A_3_ adenosine receptors [60].

Novel 3-phenylcoumarins are extracted from natural sources, characterized, and tested for versatile uses. For example, 3-phenylcoumarin extracted from *Machaerium acutifolium* reminds greatly compounds presented here with hydroxyl and methoxy substituents and has shown larvicidal activity against *Aedes aegypti* [61]. Similarly, novel 3-phenylcoumarins extracted from *Glycyrrhiza uralensis* or *Psoralea corylifolia* have been utilized for the activation of nuclear factor erythroid 2-related factor 2 [62] or to inhibit protein kinase activity and induce apoptotic cell death [63], respectively.

## 3. Figure Preparation

Coumarin compounds were drawn with 2D Sketcher in Maestro package (Schrödinger Release 2021-2: Maestro, Schrödinger, LLC, New York, NY, USA, 2021) and converted to 3D structures, including possible tautomers and protonation states at pH 7.4 ± 0.0, with LigPrep in Maestro 2021-2 (Schrödinger Release 2021-2: LigPrep, Schrödinger, LLC, New York, NY, USA, 2021). The X-ray crystal structure for human ERα (PDB: 1ERE [12] and 3ERT [13]), HSD1 (1A27 [16] and 3HB5 [17]), and aromatase (3EQM [22]) were retrieved from the RCSB Protein Data Bank (PDB; www.rcsb.org) [64,65]. Protein preparation was executed with Protein Preparation Wizard [66] (Schrödinger Release 2021-2: Protein Preparation Wizard; Epik, Impact, Prime, Schrödinger, LLC, New York, NY, USA, 2021). In protein preparation, missing side chains were added using Prime, protonation, and metal charge states for cofactors and metals were generated using Epik at pH 7.4 ± 0.0, hydrogen bonds were assigned PROPKA at pH 7.4, water molecules beyond 3 Å from heteroatoms, and finally, only hydrogens were minimized. Coumarins were docked flexibly utilizing Glide [67,68] (Schrödinger Release 2021-2: Glide, Schrödinger, LLC, New York, NY, USA, 2021). In Glide-docking, the SP mode was selected. Otherwise, default settings were utilized. 2D structures for Figure 1, Figure 3, Figure 4, Figure 6, Figure 8 and Figure 9B–D were prepared by using 2D Sketcher in Maestro package (Schrödinger Release 2021-2: Maestro, Schrödinger, LLC, New York, NY, USA, 2021). Figure 5, Figure 7, Figure 9A and Figure 10 were prepared with Bodil [69], MolScript v2.1.2 [70], and Raster3D package [71].

## 4. Conclusions

In this work, simple, cheap and easy-to synthesize 3-phenylcoumarin derivatives are shown to be utilized as steroid mimics in steroid hormone biosynthesis pathways and the following receptor activation. In general, the 3-phenylcoumarin ring system is likely to pack similarly to the hydrophobic core of steroidal compounds at the active site of steroid hormone enzymes and receptors. Moreover, a number of polar substituents (mainly hydroxyl, methoxy, or halogen) in the 3-phenyl ring R1–R3 positions and in the coumarin ring R4-R6 positions enable fine-tuning strong binding interactions and selectivity.

ER antagonists are routinely used to treat ER-positive breast cancer. Generally, the best ER ligands have two hydroxyl groups linked by a lipophilic scaffold, which places them approximately at a distance of 11 Å [11]. One of these hydroxyls forms a strong hydrogen bond network with Glu353, Arg394, and a water molecule, in the area where the hydroxyl of the phenolic A-ring of E2 binds. In the other end, the hydroxyl group mimics the 17β-hydroxyl group of E2 and forms an additional hydrogen bond with residue His524 in the E2 D-ring pocket. The molecular basis for the activity of these 3-phenylcoumarin analogs advises that the coumarin scaffold must have R2 and/or R5-functional group, such as hydroxyl moiety, to produce the antagonist effect against ERα.

Having polar substituents in the R1 and/or R2 positions in the 3-phenyl ring is critical for establishing the 3-phenylcoumarin binding and inhibition with HSD1. Adding yet another polar group at the R6 position in the coumarin ring improves the HSD1 inhibition even further, i.e., the best HSD1 inhibitors are either dual hydroxyls otherwise able to accept and/or donate a hydrogen bond in both ends of the compound. These compounds can form hydrogen bonds in the catalytic site with Ser143 and/or Tyr156 and in the other end with His222 and Glu283. In addition, in the best case, Ser223 and Tyr219 in the middle of the binding bind to the C2-carbonyl of the coumarin core. 

In short, coumarins are extensively studied compounds with great structural and pharmacological versatility. A coumarin core can be tailored with specific ring and polar moiety substitutions to bind and block different enzymes or receptors.

## Figures and Tables

**Table 1:** Inhibitory profiles of the 3-phenylcoumarin compounds *.

ID	ER Inhibition	ER Binding	HSD1 Inhibition	HSD1 Inhibition	HSD2 Inhibition
	% (10 μM)	pIC50	% (1 μM)	pIC50	% (1 μM)
**1**	59	5.5 ± 0.1 [7,8]	18 [8]	N/I [8]	37 [8]
**2**	98	N/B [8]	69 [8]	6.2 ± 0.1 [8]	7 [8]
**3**	19	N/A	N/A	N/A	N/A
**4**	19	N/A	49	N/A	N/A
**5**	101	6.5 [7]	N/A	N/A	N/A
**6**	71	N/A	13	N/A	N/A
**7**	91	5.9 ± 0.1 [7,8]	3 [8]	N/I [8]	N/A
**8**	101	6.1 ± 0.1 [7,8]	1 [8]	N/I [8]	N/A
**9**	96	6.5 [7]	N/A	N/A	N/A
**10**	0	N/B [8]	68 [8]	6.3 ± 0.2 [8]	27 [8]
**11**	N/A	N/B [8]	84 [8]	6.8 ± 0.1 [8]	16 [8]
**12**	0	N/A	20	N/A	N/A
**13**	74	N/A	5	N/A	N/A
**14**	0	N/B [8]	47 [8]	5.9 ± 0.0 [8]	42 [8]
**15**	86	6.2 ± 0.1 [8]	23 [8]	5.4 ± 0.1 [8]	31 [8]
**16**	55	N/B [8]	39 [8]	5.8 ± 0.1 [8]	13 [8]
**17**	0	N/A	4	N/A	N/A
**18**	0	N/A	4	N/A	N/A
**19**	0	N/A	4	N/A	N/A
**20**	0	N/A	11	N/A	N/A
**21**	1	N/A	1	N/A	N/A
**22**	1	N/A	0	N/A	N/A
**23**	N/A	N/A	21	N/A	N/A
**24**	57	N/A	0	N/A	N/A
**25**	9	N/A	33	N/A	N/A
**26**	0	N/A	12	N/A	N/A
**27**	N/A	N/A	5	N/A	N/A
**28**	N/A	N/A	15	N/A	N/A
**29**	N/A	N/A	32	N/A	N/A
**30**	N/A	N/A	2	N/A	N/A

N/A = not available; N/B = no binding; N/I = no inhibition. * Not marked data taken from [9].

## Abbreviations

CYP, cytochrome P450; E1, estrogen; E2, 17β-estradiol; ER, estrogen receptor; ERα, estrogen receptor α; ERβ, estrogen receptor β; HSD1, 17β-hydroxysteroid dehydrogenase type 1; HSD2, 17β-hydroxysteroid dehydrogenase type 2; MAO-A, monoamine oxidase A; MAO-B monoamine oxidase B; SERM, selective estrogen receptor modulator.
